# Interleukin-17A upregulates receptor activator of NF-κB on osteoclast precursors

**DOI:** 10.1186/ar2936

**Published:** 2010-02-18

**Authors:** Iannis E Adamopoulos, Cheng-chi Chao, Richard Geissler, Drake Laface, Wendy Blumenschein, Yoichiro Iwakura, Terrill McClanahan, Edward P Bowman

**Affiliations:** 1 Discovery Research, Schering-Plough Biopharma (formerly DNAX Research, Inc.), 901 South California Avenue, Palo Alto, CA 94304, USA; 2 Schering Plough Research Institute, 144 State Route 94, Lafayette, NJ 07848, USA; 3 Institute of Medical Science, University of Tokyo, 4-6-1 Shirokanedai, Minato-ku, Tokyo 108-8639, Japan

## Abstract

**Introduction:**

The interaction between the immune and skeletal systems is evidenced by the bone loss observed in autoimmune diseases such as rheumatoid arthritis. In this paper we describe a new mechanism by which the immune cytokine IL-17A directly affects osteoclastogenesis.

**Methods:**

Human CD14^+ ^cells were isolated from healthy donors, cultured on dentine slices and coverslips and stimulated with IL-17A and/or receptor activator of NF-κB ligand (RANKL). Osteoclast differentiation was evaluated by gene expression, flow cytometry, tartrate-resistant acid phosphatase staining, fluorescence and electron microscopy. Physiologic bone remodelling was studied in wild-type (Wt) and IL-17A^-/- ^mice using micro-computer tomography and serum RANKL/osteoprotegerin concentration. Functional osteoclastogenesis assays were performed using bone marrow macrophages isolated from IL-17A^-/- ^and Wt mice.

**Results:**

IL-17A upregulates the receptor activator for NF-κB receptor on human osteoclast precursors *in vitro*, leading to increased sensitivity to RANKL signalling, osteoclast differentiation and bone loss. IL-17A^-/- ^mice have physiological bone homeostasis indistinguishable from Wt mice, and bone marrow macrophages isolated from these mice develop fully functional normal osteoclasts.

**Conclusions:**

Collectively our data demonstrate anti-IL-17A treatment as a selective therapeutic target for bone loss associated with autoimmune diseases.

## Introduction

The pathologic outcome of the activated immune system interacting with the skeletal system is evidenced by articular bone erosion and joint loss of function seen in autoimmune joint diseases, such as rheumatoid arthritis (RA). Pathological bone resorption is the result of increased differentiation and/or activity of osteoclasts [1]. Osteoclast precursors are present in the circulating monocyte population [2], and these cells differentiate into multinucleated osteoclasts in the presence of macrophage-colony stimulating factor (M-CSF) and receptor activator for NF-κB ligand (RANKL) produced by osteoblasts [3]. Osteoprotegerin (OPG) is a soluble decoy receptor for RANKL that inhibits osteoclast formation and bone resorption [4]. Terminally differentiated osteoclasts polarize onto the bone surface by forming filamentous actin (F-actin) rings associated with α_v_β_3 _integrin, acidify the enclosed extracellular space using a proton pump [5] to solubilize the inorganic calcium phosphate, and release organic matrix-degrading enzymes such as cathepsin K, matrix metalloproteinases and tartrate-resistant acid phosphatase (TRAP) that results in a continuous series of resorption lacunae on the bone. The receptor activator for NF-κB (RANK)/RANKL/OPG axis governs homeostatic bone remodelling, as mice deficient in RANK, RANKL, or OPG have severe bone phenotypes [3,4,6].

In addition to producing RANKL, activated T cells produce other proinflammatory factors, such as TNF, which stimulate osteoclastogenesis in RANK^-/- ^mice [6] and induce osteoclastogenesis *in vitro *by direct stimulation of murine bone-marrow-derived macrophages exposed to permissive levels of RANKL [7]. Haematopoietic precursors from RANKL^-/-^, RANK^-/-^, or TRAF6^-/- ^mice also become osteoclasts *in vitro *when they are stimulated with TNFα in the presence of cofactors such as transforming growth factor beta [8], and recently TNF was shown to induce osteoclastogenesis from spleen-cell-derived macrophages in the absence of RANKL [9]. Collectively these data demonstrate that immune-mediated osteoclastogenesis pathways can lead to joint pathology in inflammatory arthritis in both a RANKL-dependent and a RANKL-independent manner [10-12].

The IL-23/IL-17A axis has recently been implicated in joint pathology and autoimmune diseases. The proinflammatory cytokine IL-23 promotes the differentiation of a novel memory T-cell subset (Th17) in mice characterized by the production of the signature cytokine IL-17A. IL-23 is comprised of the IL-23p19 subunit and the IL-12p40 subunit, (shared with IL-12), and IL-23p19^-/- ^mice have reduced IL-17A^+^-Th17 cells, are resistant to collagen-induced arthritis induction, and have no joint or bone pathology [13]. Th17 cells stimulate local inflammation and express RANKL as well as inducing RANKL expression in osteoblasts [14,15].

Since both Th17 cells and osteoclast precursors are present in the peripheral blood of healthy adults and both are elevated in the synovial fluid of RA patients [2,14,16], we studied the role of IL-17A in the direct differentiation and activation of osteoclast precursors [17]. Specifically, we analysed the *in vitro *mechanisms by which IL-17A affects osteoclastogenesis, using mice deficient for IL-17A, and treating human peripheral blood monocytes with exogenous cytokines. We confirm that IL-17A does not play a role in physiological bone homeostasis, and show that IL-17A sensitizes osteoclast precursors to the key osteoclast factor RANKL by increasing RANK expression on osteoclast precursors [18]. These data provide a direct mechanism of IL-17A action on osteoclast precursors that is distinct from IL-17A's known action on osteoblasts [14], thereby providing an additional link between the immune and skeletal systems. Furthermore, the induction of RANK and RANKL expression make IL-17A a potent inducer of bone erosion under inflammatory conditions and its blockade may be used to combat disabling conditions such as RA.

## Materials and methods

### Reagents and antibodies

All cell incubations were performed in culture medium consisting of α-minimal essential medium (Invitrogen, San Francisco, CA, USA), 2 mM glutamine, 10% heat-inactivated foetal bovine serum (Invitrogen), 100 IU/ml penicillin, and 100 μg/ml streptomycin. Human soluble RANKL was detected in the conditioned medium using a sandwich ELISA (Biovendor, Candler, NC, USA), and mouse RANKL and OPG was measured in the serum of 8-week-old mice using ELISA from R&D Systems (San Francisco, CA, USA).

### Mice and mouse osteoclast cultures

All animal protocols were approved by Schering-Plough Biopharma's Institutional Animal Care and Use Committee. IL-17A^-/- ^mice have been previously described [19]. The mice were sacrificed by carbon dioxide exposure and blood was collected by cardiac puncture. Whole bone marrow was extracted from the tibia and femurs of 6-week-old to 8-week-old wild-type (Wt) C57Bl/6 and IL-17A^-/- ^mice. Cells were plated in culture medium containing 25 ng/ml M-CSF. Osteoclasts were generated in 5-day cultures of bone marrow macrophages with 25 ng/ml M-CSF and 50 ng/ml soluble RANKL.

### Micro-computer tomography

Mouse femurs were stored in 10% neutral buffered formalin at 2 to 8°C until processing. Samples were scanned at Numira (Salt Lake City, UT, USA) using a high-resolution, volumetric μCT40 scanner (Scanco Medical AG, Basserdorf, Switzerland). The image data were acquired at 6 μm isometric voxel resolution with 300 ms exposure time, 2,000 views, and five frames per view. The micro-computer tomography-generated DICOM files were used to analyse the samples and to create volume renderings of the region of interest. The raw data files were viewed using Microview (GE Healthcare, Milwaukee, WI, USA).

Utilizing ScanCo Medical software, bone density measurements were obtained for the distal end of the femur and the midshaft. For distal femur analysis, a three-dimensional trabecular volume was selected 0.5 mm below the growth plate and 0.5 mm thick. For midshaft analysis, the length of the entire femur was measured and a 1 mm thick mid-cortical section was used for analysis. A threshold of 20% of the 16-bit total grey-scale values between 0 and 32,000 was used. Three-dimensional image rendering were generated through original volumetric reconstructed images using Microview software (GE Healthcare, Piscataway, NJ, USA).

### Human osteoclast cultures

Buffy coats were obtained from normal healthy human volunteers participating in the Stanford Medical School Blood Center blood donation programme, with the permission of the institution and volunteers, in accordance with the Declaration of Helsinki. Human protocols were approved by the Schering Plough Institutional Biosafety Committee, which acts as an ethical committee for the approval of studies involving the use of human tissue.

Peripheral blood mononuclear cells were isolated by gradient density centrifugation with Histopaque-1077 (Sigma-Aldrich, St Louis, MO, USA) as previously described [20]. CD14^+ ^cells were isolated with the MACS monocyte isolation Kit (Miltenyi Biotech Auburn, CA, USA). The purity of the isolated CD14^+ ^cells was >95% as assessed by flow cytometry at day 1. Peripheral blood mononuclear cells (PBMCs) or CD14^+^cells were added to 96-well tissue culture plates containing dentine slices (Nordic Bioscience Diagnostics, Herlev, Denmark) or coverslips (Electron Microscopy Sciences, Hatfield, PA, USA) as previously described [20]. PBMC cultures were maintained in the presence of 30 ng/ml soluble RANKL and 25 ng/ml M-CSF as positive control. PBMC cultures were maintained with M-CSF alone as a negative control. All PBMC cultures were incubated for up to 21 days, during which time the entire culture medium containing all factors was replenished every 2 or 3 days.

### Cytochemical and functional assessment of osteoclast formation

#### Tartrate-resistant acid phosphatase

The cells cultured on plastic dishes were stained for TRAP using a commercial kit (387-A; Sigma) according to the manufacturer's instructions.

#### F-actin ring

To detect the F-actin ring structure [21], dentine slices were fixed with 4% formaldehyde for 5 minutes and then permeabilized for 6 minutes in 0.5% Triton X-100 (in PBS) and rinsed with PBS. The cells on dentine slices were then incubated with 0.1 μM TRITC-conjugated phalloidin (Sigma-Aldrich, St Louis, MO, USA) for 30 minutes, washed and rinsed with PBS before mounting with DAPI (Vectashield; Vector Peterborough, UK), and were observed using a fluorescence microscope (Nikon, Melville, NY, USA).

#### Resorption assay

Functional evidence of osteoclast formation was determined by a lacunar resorption assay system using cell cultures on dentine slices as previously described [20]. Cells were removed from the dentine slices by treatment with 0.1 M ammonium hydroxide. The dentine slices were washed in distilled water and ultrasonicated to remove adherent cells, then stained with 0.5% (v/v) toluidine blue to reveal areas of lacunar resorption and examined by light microscopy.

### Scanning electron microscopy

Cells on dentine were fixed in 4% glutaraldehyde, dehydrated by passing through graded alcohols and then through graded (50 to 100%) hexamethyl-disilazane solution (Sigma-Aldrich) before being air-dried. Dentine slices were then mounted onto aluminium stubs (EMS, Hatfield, PA, USA), sputtered with gold, and examined using a Philips SEM 505 scanning electron microscope.

### RNA extraction and real-time quantitative PCR

Total RNA was purified from different stages of osteoclast cultures using the RNeasy Mini Kit (QIAGEN, Valencia, CA, USA). Gene expression was calculated using the Δ-ΔCt method (using the mean cycle threshold Ct value for ubiquitin and the gene of interest for each sample). The equation used to obtain the normalized values was:

### Flow cytometry of isolated bone marrow macrophages, PBMCs and CD14^+ ^cells

Bone marrow cells were flushed from the femurs and tibia of C57BL/6 AnN mice (Taconic, Oxnard, CA, USA) and IL-17A^-/- ^mice and were dispersed to single-cell suspensions. Nonspecific binding was blocked by pretreating cells with rat anti-mouse CD16/32 mAb (BD Biosciences, San Jose, CA, USA) for 10 minutes at room temperature. Alexa Fluor^® ^647-conjugated rat anti-mouse CD11b mAb, PE-conjugated rat anti-mouse Gr-1 mAb and isotype controls were all obtained from BD Biosciences. Cells were stained using predetermined optimized mAb concentration, and cell surface phenotyping was done using a BD FACS Caliber flow cytometer (BD Biosciences) and analysed using FlowJo software (Tree Star, Ashland, OR, USA).

PBMCs were analysed for the expression of c-fms and RANK receptors on CD14^+ ^cells. Briefly, cells were resuspended in PBS, incubated with mouse anti-human antibody for 30 minutes at 4°C, washed with PBS, and fixed with 1% formaldehyde. Expression of receptors was determined by staining cells with anti-human CD14-APC and c-fms-Biotin and/or RANK-PE. Gated events (100,000) were collected using the FACScan system and were analysed with CELLQuest software (Becton Dickinson, Franklin Lakes, NJ, USA).

### Statistical analysis

Human data were analysed by Kruskal-Wallis test with Dunn's multiple-comparison after test. One-way or two-way analysis of variance with Bonferroni after test was used where appropriate. *P *< 0.05 was considered statistically significant (n = 3, unless otherwise indicated).

## Results

### IL-17A induces human osteoclast formation

**Figure 1:**
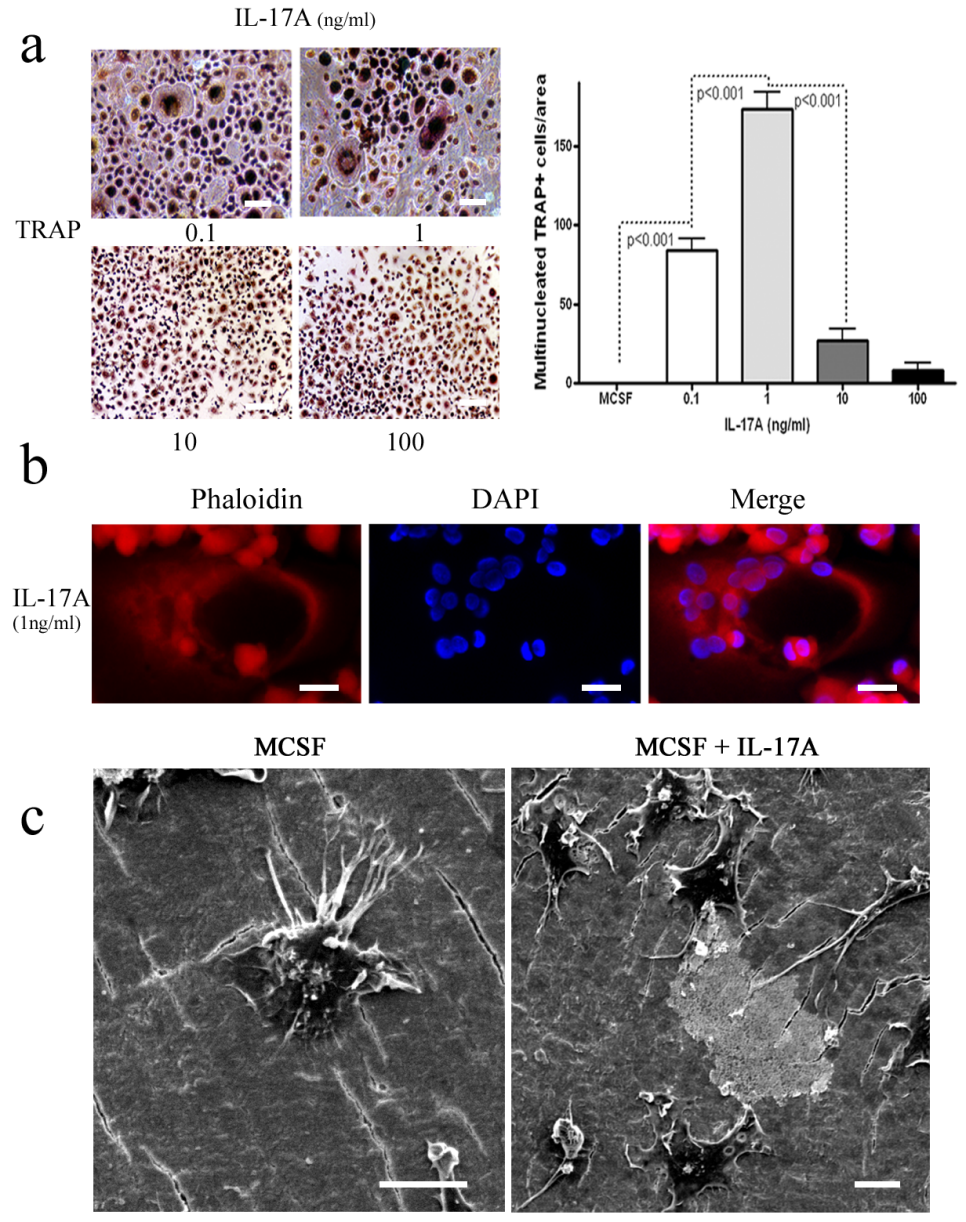
**IL-17A induces low-grade resorption**. **(a) **Tartrate-resistant acid phosphatase (TRAP) staining of peripheral blood mononuclear cells isolated from healthy volunteers cultured for 14 days in the presence of macrophage colony-stimulating factor (MCSF) and increasing IL-17A concentrations. Data pooled from three individual experiments performed in triplicate. **(b) **Phaloidin and DAPI staining of CD14^+ ^cells treated with IL-17A (1 ng/ml). **(c) **Scanning electron photomicrographs of CD14^+ ^cells cultured for 21 days treated with IL-17A (1 ng/ml) on dentine slices in the presence of MCSF (25 ng/ml). Bars: left, 20 μm; right, 40 μm. Representative data of three individual experiments.

**Figure 2:**
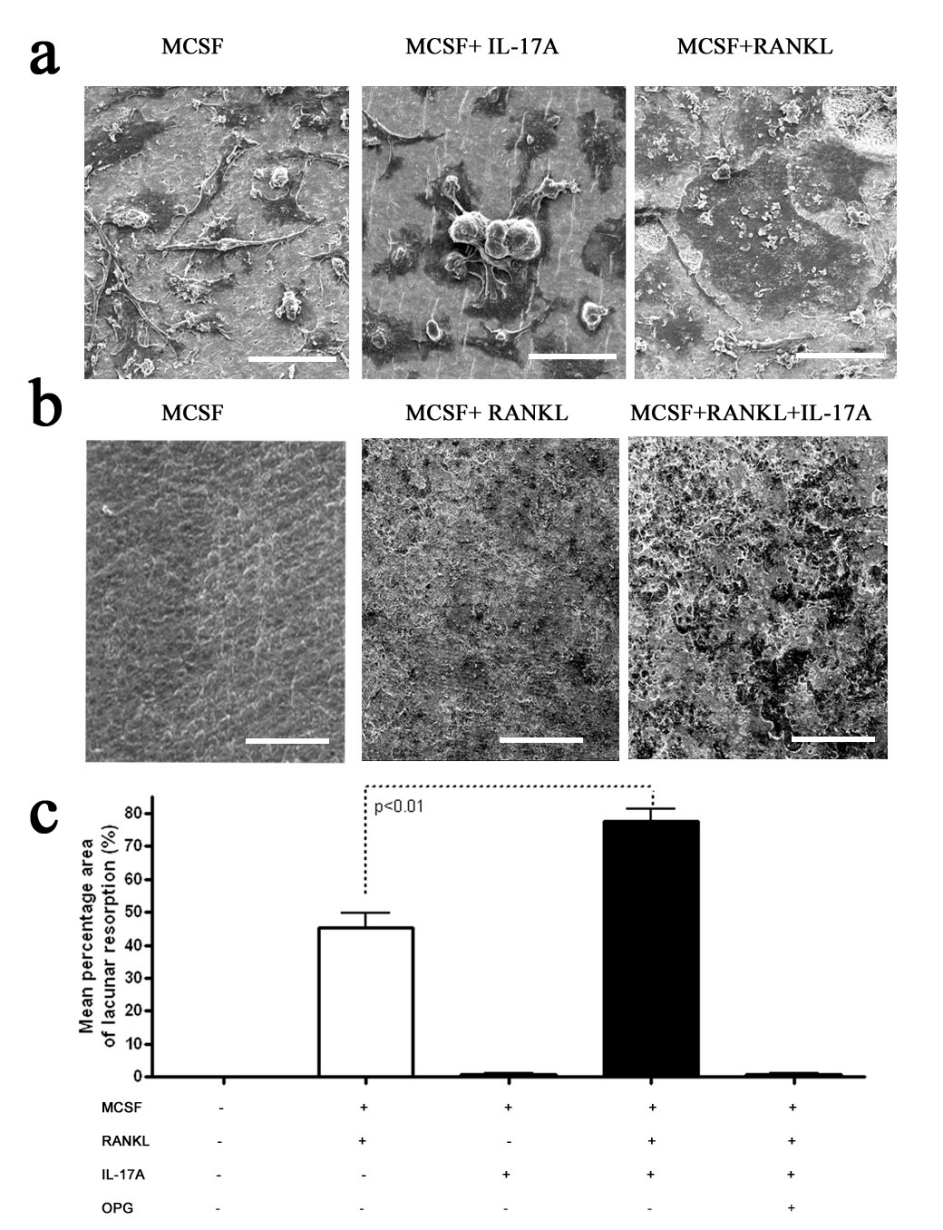
**IL-17A increases bone resorption in synergy with receptor activator of NF-κB ligand**. CD14^+ ^cells cultured on dentine slices for 18 days in the presence of macrophage colony-stimulating factor (MCSF) and receptor activator of NF-κB ligand (RANKL) and/or IL-17A showing **(a) **increased cell fusion and **(b) **increased bone resorption. Bars (left to right): upper panel, 45, 45 and 60 μm; lower panel, 70, 100 and 100 μm. **(c) **Mean percentage dentine erosion of control versus IL-17A-stimulated CD14^+ ^cells, *P *< 0.01. Data pooled from four individual experiments performed in triplicate.

Exogenous IL-17A stimulation of human osteoclast precursors within the PBMC population stimulated the formation of large, TRAP^+^, multinucleated cells in the absence of exogenous RANKL stimulation (Figure 1a). Very low IL-17A concentrations were needed to induce TRAP^+ ^cells (0.1 to 1 ng/ml) - concentrations much lower than needed to drive maximal proinflammatory cytokine and chemokine expression by endothelial and mesenchymal cells (unpublished data). The TRAP^+ ^multinucleated cells (30 to 40 μm) formed by culturing human osteoclast precursors with IL-17A did form F-actin rings and showed an uncommon low-grade dentine resorption (Figure 1b, c). Low-grade resorption was also associated with smaller (<20 μm) mononuclear cells in the IL-17A-treated cultures, but these cells also failed to form lacunar resorption formation typically seen by fully mature RANKL-derived osteoclasts. IL-17A stimulation increased cell fusion giving rise to multinucleated cells in the culture, but the cell fusion did not give rise to the same distinctive multinucleated giant cell that arise in RANKL-treated cultures (Figure 2a, upper panel). These data above address whether IL-17A drives osteoclastogenesis independently of RANKL but in the presence of M-CSF.

We next addressed whether IL-17A had a synergistic effect with RANKL in driving osteoclastogenesis. Human osteoclast precursors cultured with RANKL differentiate into multinucleated osteoclasts capable of forming F-actin rings and lacunar resorption (Figure 2a, lower panel). The addition of 1 ng/ml IL-17A to the RANKL-treated cultures increased the mean area of dentine resorbed by as much as 30% more (Figure 2b).

### IL-17A upregulates RANK and c-fms on osteoclast precursors

**Figure 3:**
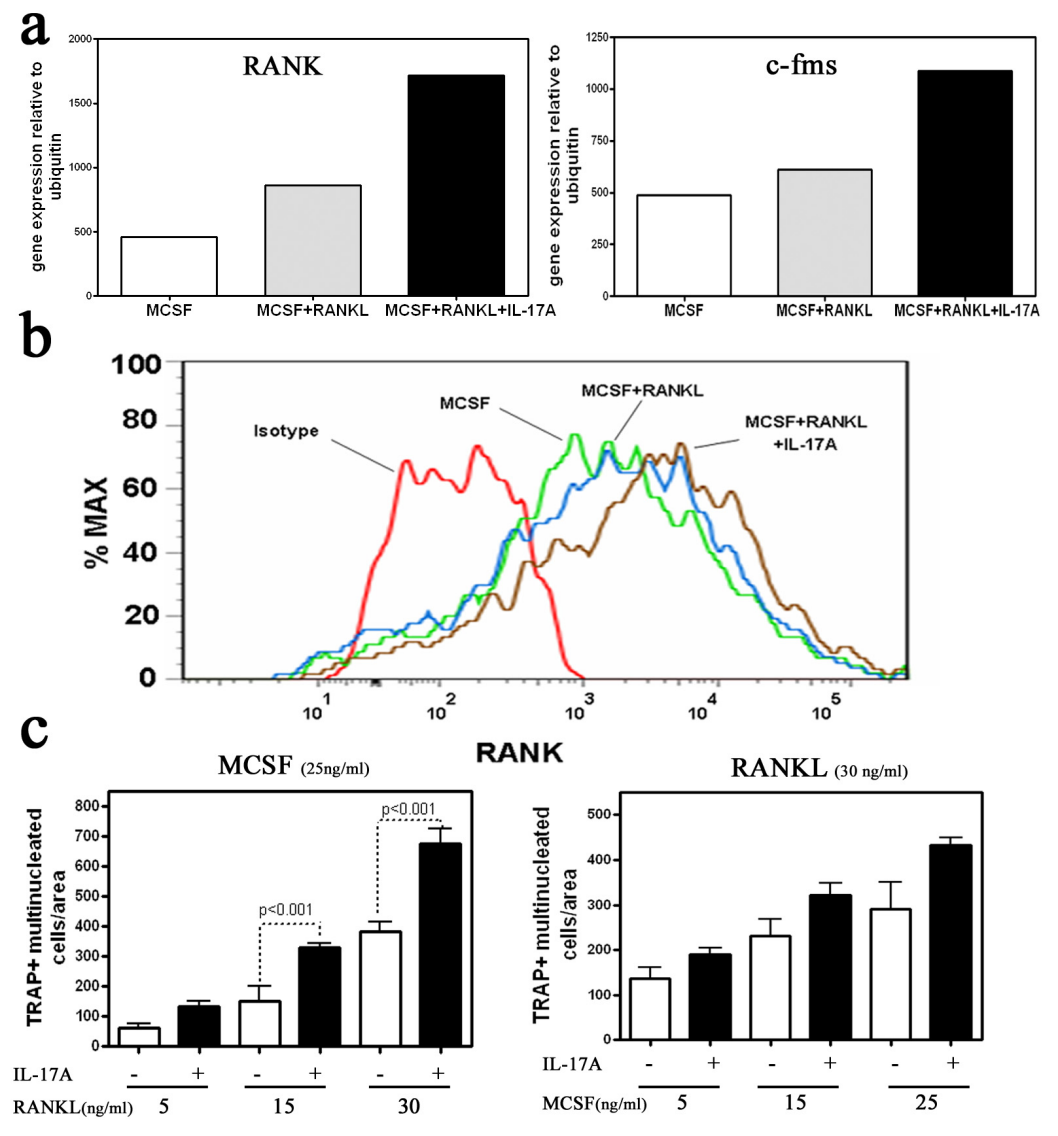
**IL-17A sensitizes pre-osteoclasts to the receptor activator of NF-κB ligand signal**. **(a) **CD14^+ ^cells' representative m-RNA expression of receptor activator of NF-κB (RANK) and c-fms receptor from one donor. Representative data of five donors. **(b) **Surface RANK expression after treatment with macrophage colony-stimulating factor (MCSF) and/or receptor activator of NF-κB ligand (RANKL) and/or IL-17A. Representative data of two experiments. **(c) **Mean number of tartrate-resistant acid phosphatase (TRAP)^+ ^multinucleated cells in the presence of IL-17A across a gradient of RANKL whilst keeping MCSF constant (left) and across a MCSF gradient whilst keeping RANKL constant (right). Data pooled from two experiments.

It was unexpected that IL-17A could synergize with RANKL under inflammatory conditions to drive increased bone resorption. Forty osteoclast-related genes were evaluated by quantitative PCR to uncover the mechanism by which IL-17A synergizes with RANKL to induce osteoclastogenesis. The increased bone resorption, correlated with increased TRAP expression (data not shown), as well as with increased RANK and c-fms expression in cultured cells (Figure 3a). An increase in TRAP expression was expected, but did not provide a mechanistic clue of how IL-17A synergized with RANKL. Elevated RANK message was confirmed by flow cytometry of osteoclast precursors showing increased surface RANK protein expression following addition of IL-17A to the differentiation culture (Figure 3b). Surface expression of the M-CSF receptor c-fms could not be confirmed by flow cytometry (data not shown), suggesting either that the extensive trypsin treatment to suspend cells degraded the molecule to a form no longer recognized by the detection antibody or that the expression was below the limits detected by flow cytometry.

IL-17A-elevated c-fms and RANK expression on human osteoclast precursors was hypothesized to sensitize the osteoclasts precursors to M-CSF and RANKL signalling, resulting in greater osteoclast number and bone resorption under limiting M-CSF or limiting RANKL conditions. By keeping optimal M-CSF levels constant and decreasing the RANKL concentration (and *vice versa*) while stimulating with IL-17A, we demonstrated that IL-17A stimulation resulted in an increase of multinucleated TRAP^+ ^cell formation under limiting M-CSF and RANKL conditions (Figure 3c).

### IL-17A^-/- ^mice have normal bone homeostasis and develop normal, fully functional osteoclasts

**Figure 4:**
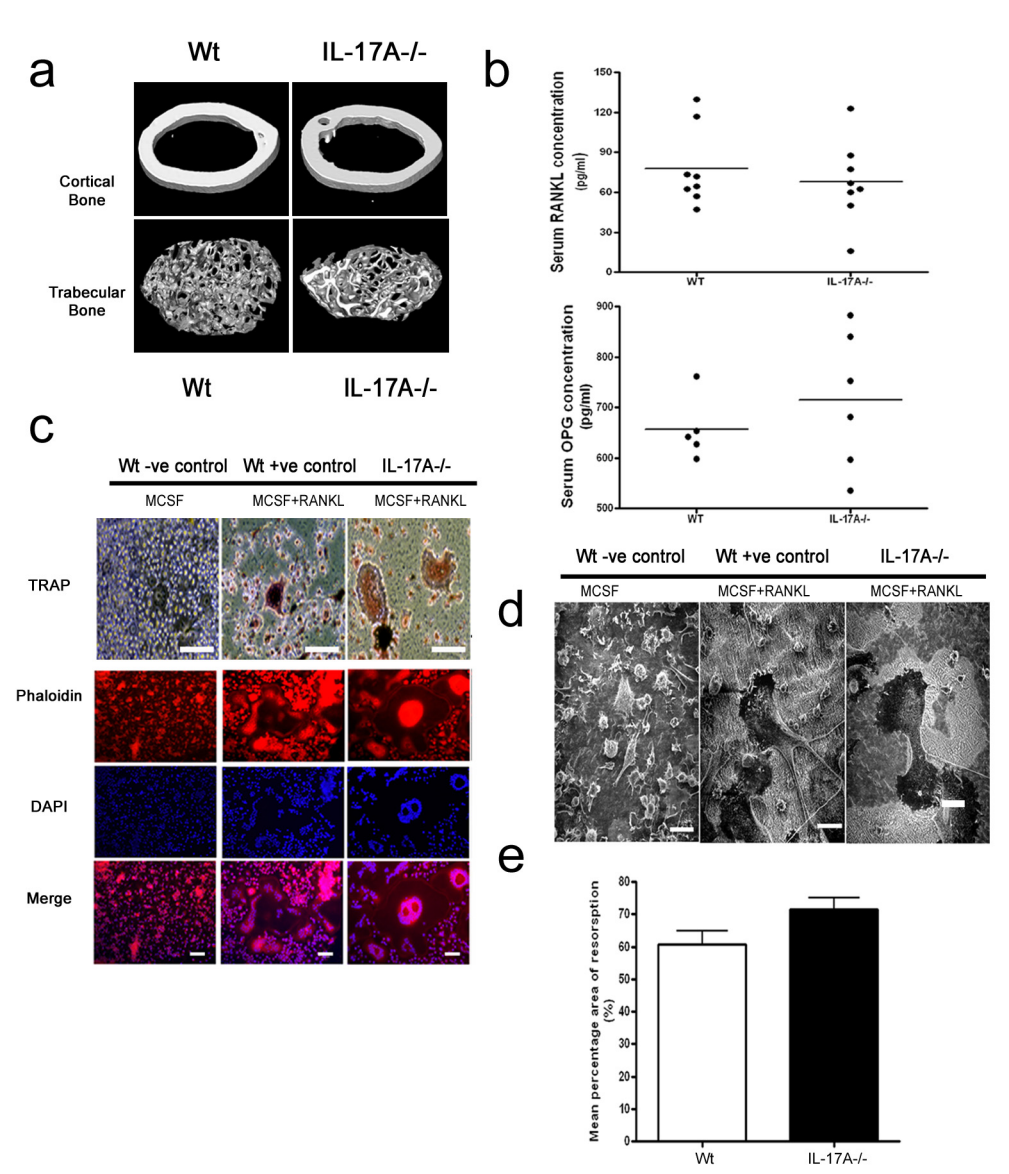
**IL17A^-/- ^mice have normal bone mineral densities and osteoclast formation**. **(a) **High-resolution micro-computer tomography analysis of 8-week-old male mouse femur midshaft and distal trabecular bone from IL-17A and wild-type (Wt) mice. **(b) **Serum receptor activator of NF-κB ligand (RANKL)/osteoprotegerin (OPG) levels of 8-week-old to 10-week-old IL-17A^-/- ^mice and control mice. **(c) **Upper: bone marrow macrophages isolated from IL-17A^-/- ^mice cultured for 6 days in the presence of macrophage colony-stimulating factor (MCSF)and RANKL form multinucleated tartrate-resistant acid phosphatase (TRAP)^+ ^cells. Bars: 50 μm. Lower: Phalloidin, DAPI staining, and merged image of both stains of bone marrow macrophages isolated from IL-17A^-/- ^mice and control mice cultured for 6 days in the presence of MCSF and RANKL showing F-actin ring formation. Bars: 25 μm. **(d) **Scanning electron photomicrographs of BMM cultures showing mature osteoclast resorbing activity (resorbed dentine has a rough, lighter colour appearance). Bars: 50 μm. Representative data of three experiments performed in triplicate. **(e) **Mean percentage area of dentine resorption of IL-17A and Wt mice.

The bone mineral density of cortical and trabecular bone was not significantly different between Wt mice and IL-17A^-/- ^mice (Figure 4a). Serum OPG and RANKL levels in IL-17A^-/- ^mice were similar to Wt mice (Figure 4b). The bone marrow isolated from IL-17A^-/- ^mice had equal numbers of CD11b^+ ^cells (osteoclast precursors) (Figure 4c), and when macrophages were stimulated with M-CSF and RANKL they differentiated into multinucleated TRAP^+ ^cells capable of F-actin ring formation and dentine resorption (Figure 4d, e) - thus fulfilling the functional characteristics of osteoclasts. No differences were observed in any stage of osteoclast differentiation, nor in the function or activity of mature cells, and quantitative gene expression analysis confirmed that all osteoclast-related genes were similarly upregulated in *in vitro *differentiation cultures from IL-17A^-/- ^mice compared with Wt mice (data not shown).

These *in vivo *and *in vitro *data support the notion that IL-17A is not required for normal skeletal development and physiological osteoclast activity. IL-17A would only be expressed in the bone microenvironment during times of host defence in the bone (for example, septic arthritis) or during an autoimmune joint disease (for example, RA).

## Discussion

IL-17A is the signature cytokine of the recently discovered Th17 memory T-cell subset. Th17 cells are the only T-cell subtype that express RANKL [15], which is the main osteoclast differentiation factor leading to the differentiation of osteoclast precursors to mature bone resorbing osteoclasts [3]. IL-17A-producing Th17 cells are present in the joints of RA patients [22,23] and synovial membrane IL-17A gene expression was one factor that was predictive for subsequent bone erosion and joint damage [24]. IL-17A is present in RA synovial fluid [14], and RA synovial fluid macrophages can differentiate to fully functional osteoclasts [16]. In the collagen-induced arthritis model, IL-17A^-/- ^mice are protected from joint disease with less TRAP^+ ^cells correlating with reduced bone resorption, suggesting that IL-17A plays a role in osteoclastogenesis [25,26]. IL-17A can act independently of TNF under arthritic conditions [27] and stimulates cartilage destruction in the IL-1-deficient mice [28], supporting the hypothesis that IL-17A can act independently of IL-1 [29]. Moreover, the combination of IL-17A with IL-1 and TNF shows a marked increase in inflammation and bone destruction [30,31]. Collectively the above experiments elegantly show that IL-17A acts independently of IL-1 and TNF but can also synergize with those factors.

The direct link of IL-17A to osteoclastogenesis, however, remained unknown. In this manuscript we focused on IL-17A's direct role in the regulation of osteoclastogenesis in inflammatory arthritis. To investigate how pathologic IL-17A expression could impact bone remodelling we studied the effects of exogenously added IL-17A on PBMCs and CD14^+ ^cells. As mentioned previously IL-17A acts on stromal cells to induce the expression of RANKL. To eliminate this component of IL-17A biology we employed a culture system where there are no stromal cells but only CD14^+ ^cells. Moreover, no RANKL was detected in the conditioned medium of CD14^+ ^cells stimulated with 1, 10, 50 and 100 ng/ml IL-17A after 1 to 24 hours.

IL-17A stimulated the formation of multinucleated TRAP^+ ^from PBMC cultures at concentrations as low as 1 ng/ml (Figure 1a). In order to study the direct effect of these cytokines on osteoclast precursors, we used the CD14^+ ^fraction of PBMCs and confirmed that IL-17A induced TRAP^+^multinucleated cells. The use of TRAP^+ ^cells as an osteoclast marker has been overrated in recent literature, especially since macrophage polykaryons, immature dendritic cells, and mononuclear macrophages all stain positive for TRAP [15,16,32-34]. Osteoclast functional assays were therefore performed to validate the findings. IL-17A-treated CD14^+ ^cells formed F-actin rings and induced low-grade resorption, but notably did not induce lacunar excavation as in the RANKL-treated cultures (Figure 1b, c).

Cell morphology was studied by scanning electron microscopy. Cultures treated with IL-17A contained numerous small cells (<30 μm) rounded or flattened and spread over the dentine surface, to which they were attached by fine microvilli. Some cells had cytoplasmic processes that extended up to 20 μm over the dentine surface. These cells had numerous surface ruffles and were able to perform low-grade resorption (Figure 1c). The area of low-grade resorption was not similar to the lacunar excavation observed in RANKL-treated cultures and was generally small, discrete, round or ovoid areas that did not possess a well-defined margin and did not coalesce to form large areas of lacunar excavation (Figure 1c).

Moreover, IL-17A-stimulated CD14^+ ^cell fusion was distinct from the cell fusion observed in RANKL-treated cultures, which formed uniform multinucleated giant cells (Figure 2a). Cell fusion in IL-17A-treated cultures was incomplete, with cell membranes only partly coalesced together and individual cells being distinguishable (Figure 2a). The exact mechanism of IL-17A on cell fusion remains to be elucidated; however, it is noteworthy that IL-17A induces dendritic cell fusion [35]. IL-17A addition to monocyte-derived dendritic cells induced a semi-mature, mixed monocyte-macrophage-dendritic cell phenotype with cells expressing CD14, CD68, CD1a, MHC II and CCR6 and induced dendritic cell fusion. The dendritic cell fusion was characterized as less efficient and the number and size of nuclei was smaller (four to eight nuclei) when compared with the previously described M-CSF and RANKL fusion pathway [35]. We confirm the above data and show that cell fusion occurred in IL-17A-stimulated CD14^+ ^cells cultured for 18 days, and also also show that cells contained more than 20 nuclei as evidenced by DAPI staining (Figure 1b) and that complexes of partially fused cells were as large as 60 μm (Figures 2a).

We initially reasoned that IL-17A's low-grade resorption was due to the upregulation of matrix metalloproteinase-9 [36]; however, no significant increase in matrix metalloproteinase-9 message was detected (data not shown). More importantly IL-17A synergized with RANKL-treated cultures to increase bone resorption by 30% (Figure 2b, c). IL-17A could not synergize with TNF to increase osteoclastogenesis and its synergy with RANKL was blocked by OPG, further confirming the fact that it is a RANKL-mediated effect (Figure 2c). The only osteoclast-related genes (out of a panel of 60 genes) that were significantly upregulated were TRAP (data not shown), c-fms and RANK (Figure 3a).

RANKL addition to IL-17A-treated CD14^+^cultures showed increased bone resorption that correlated with increased RANK, as shown by flow cytometry (Figure 3b). To measure the osteoclastogenic potential of IL-17A on the c-fms and RANK receptors, we performed a quantitative TRAP assay where cells were cultured under standard concentrations of MCSF (Figure 3c, left) or RANKL (Figure 3c, right) and the addition of increasing dose of RANKL or MCSF, respectively, was tested in the presence or absence of 1 ng/ml IL-17A. Addition of IL-17A sensitized the pre-osteoclasts to both MCSF and RANKL, leading to increased osteoclastogenesis (Figure 3c).

Bone marrow macrophages from Wt mice and IL-17A^-/- ^mice were not statistically different under homeostatic conditions and the osteoclasts that develop in *in vitro *cultures function normally in their ability to form multinucleated TRAP^+ ^cells capable of actin ring formation and lacunar resorption (Figure 4). Moreover, there was no detectable modulation of physiologic bone remodelling in IL-17A-deficient mice; serum RANKL and OPG levels were similar to control mice, and no differences in cortical or trabecular bone mineral density were observed (Figure 4). These data are in agreement with previous studies where using 40 μm resolution dual-energy X-ray absorptiometry revealed no obvious abnormality in skeletal development and bone morphometric analyses, concluding that bone resorption and formation was normal in IL-17A^-/- ^mice [15].

Our findings confirm the *in vitro *observations and suggest that IL-17A has a dual effect on RANKL-induced osteoclastogenesis: firstly, IL-17A upregulates RANKL on osteoclastogenesis supporting cells; and, secondly, IL-17A upregulates RANK on pre-osteoclasts, making them hypersensitive to the RANKL signal. To the best of our knowledge the present report is the first that highlights a direct mechanism of osteoclastogenesis by IL-17A via the upregulation of RANK in osteoclast precursors.

## Conclusions

IL-17A does not play a role in physiological bone remodelling as shown by our IL-17A^-/-^-deficient mice studies; however, IL-17A is secreted by Th17 cells under inflammatory conditions. We have shown that IL-17A upregulates RANK in osteoclast precursors, sensitizing them to RANKL-induced bone resorption. We therefore propose IL-17A as a suitable target to combat bone loss in inflammatory arthritis and autoimmune diseases such as RA.

## Abbreviations

DAPI: 4',6-diamidino-2-phenylindole; F-actin: filamentous actin; IL: interleukin; mAb: monoclonal antibody; M-CSF: macrophage colony-stimulating factor; NF: nuclear factor; OPG: osteoprotegerin; PBMC: peripheral blood mononuclear cell; PBS: phosphate-buffered saline; PCR: polymerase chain reaction; RA: rheumatoid arthritis; RANK: receptor activator of NF-κB; RANKL: receptor activator of NF-κB ligand; Th17: T-helper type 17; TNF: tumour necrosis factor; TRAP: tartrate-resistant acid phosphatase; Wt: wild type.

## Competing interests

IEA, C-cC, RG, DL, WB, TM and EPB are employees of Schering-Plough Corporation and therefore receive salary and/or hold stock in Schering-Plough. YI declares that he has no competing interests.

## Authors' contributions

IEA designed and coordinated experiments, carried out all the human and mice osteoclast assays, and wrote the manuscript. C-cC performed the micro-computer tomography analysis. RG participated in the scanning electron microscopy sample preparation and analysis. WB and TM performed quantitative PCR and statistical analysis. YI and DL participated in the IL-17A^-/- ^mice studies. EPB conceived the study, designed experiments and participated in the writing of the manuscript. All authors read and approved the final manuscript.
